# Psychological Characteristics of Sex Offenders

**DOI:** 10.1192/j.eurpsy.2022.2072

**Published:** 2022-09-01

**Authors:** W. Oronowicz-Jaśkowiak, M. Lew-Starowicz

**Affiliations:** 1 Medical University of Warsaw, Medical Communication, Warsaw, Poland; 2 Centre of Postgraduate Medical Education, Department Of Psychiatry, Warsaw, Poland

**Keywords:** sexual preference disorders, paraphilic disorders, personality

## Abstract

**Introduction:**

A significant problem for clinical judicial experts when issuing court opinions is the possibility that the assessed person may be simulating, as well as lack of examination tools that would increase the objectivity and reliability of the assessment. This presentation covers studies on psychological characteristics of perpetrators of crimes against sexual freedom.

**Objectives:**

The participants were asked to complete psychological tools - Rosenberg Self-Esteem Scale, Satisfaction with Life Scale, Emotion Understanding Test, Revised NEO Personality Inventory, Attachment Style Questionnaire.

**Methods:**

The participants of the study consisted of 225 men serving sentences of imprisonment in a dozen of Polish prisons. Two clinical populations were compared: of perpetrators diagnosed and not diagnosed with sexual preference disorders. The control group consisted of offenders of crimes other than against sexual freedom.

**Results:**

no personality and psychosocial variables were identified that would significantly differentiate offenders diagnosed and not diagnosed with parapaphilic disorder.

**Conclusions:**

The results of this study justify the use of selected tools to complement the clinical diagnosis, allowing for obtaining additional data, independent from case files and interview, that would increase the probability of sexual preference disorders.

**Disclosure:**

The study was approved by the ethical committee at the Institute of Psychiatry and Neurology in Warsaw and the Director General of the Prison Service. Scientific work was financed from the budget for science in the years 2017-2021, as a research project D

